# Self-Management among Stroke Survivors in the United States, 2016 to 2021

**DOI:** 10.3390/jcm13154338

**Published:** 2024-07-25

**Authors:** Ajith Kumar Vemuri, Seyyed Sina Hejazian, Alireza Vafaei Sadr, Shouhao Zhou, Keith Decker, Jonathan Hakun, Vida Abedi, Ramin Zand

**Affiliations:** 1 Department of Neurology, College of Medicine, The Pennsylvania State University, 30 Hope Drive, Hershey, PA 17033, USA; avemuri@pennstatehealth.psu.edu (A.K.V.); sina.hej95@gmail.com (S.S.H.); jhakun@pennstatehealth.psu.edu (J.H.); 2 Department of Public Health Sciences, College of Medicine, Pennsylvania State University, Hershey, PA 17033, USA; asadr@pennstatehealth.psu.edu (A.V.S.); szhou1@pennstatehealth.psu.edu (S.Z.); vabedi@pennstatehealth.psu.edu (V.A.); 3 Department of Computer and Information Sciences, University of Delaware, Newark, DE 19716, USA; decker@udel.edu

**Keywords:** stroke, self-management, physical activity, body mass index, smoking, alcohol

## Abstract

**Background**: Self-management among stroke survivors is effective in mitigating the risk of a recurrent stroke. This study aims to determine the prevalence of self-management and its associated factors among stroke survivors in the United States. **Methods**: We analyzed the Behavioral Risk Factor Surveillance System (BRFSS) data from 2016 to 2021, a nationally representative health survey. A new outcome variable, stroke self-management (SSM = low or SSM = high), was defined based on five AHA guideline-recommended self-management practices, including regular physical activity, maintaining body mass index, regular doctor checkups, smoking cessation, and limiting alcohol consumption. A low level of self-management was defined as adherence to three or fewer practices. **Results**: Among 95,645 American stroke survivors, 46.7% have low self-management. Stroke survivors aged less than 65 are less likely to self-manage (low SSM: 56.8% vs. 42.3%; *p* < 0.0001). Blacks are less likely to self-manage than non-Hispanic Whites (low SSM: 52.0% vs. 48.6%; *p* < 0.0001); however, when adjusted for demographic and clinical factors, the difference was dissipated. Higher education and income levels are associated with better self-management (OR: 2.49, [95%CI: 2.16–2.88] and OR: 1.45, [95%CI: 1.26–1.67], respectively). Further sub-analysis revealed that women are less likely to be physically active (OR: 0.88, [95%CI: 0.81–0.95]) but more likely to manage their alcohol consumption (OR: 1.57, [95%CI: 1.29–1.92]). Stroke survivors residing in the Stroke Belt did not self-manage as well as their counterparts (low-SSM: 53.1% vs. 48.0%; *p* < 0.001). **Conclusions**: The substantial diversity in self-management practices emphasizes the need for tailored interventions. Particularly, multi-modal interventions should be targeted toward specific populations, including younger stroke survivors with lower education and income.

## 1. Introduction

In the United States, approximately 795,000 individuals experience a stroke each year, with 185,000 being recurrent cases [1]. This risk of a recurrent stroke can be addressed through effective stroke prevention strategies, including self-management and mitigation of lifestyle-related risk factors [2,3]. Self-management strategies, defined as “the active management by individuals of their treatment, symptoms, lifestyle, and the physical and psychological consequences inherent in living with a chronic condition”, are crucial in facilitating long-term behavioral changes in stroke survivors [4].

Recurrent stroke prevention encompasses the effective self-management of multiple lifestyle and medical risk factors. Despite the recognized importance of self-management, there is a lack of nationwide data concerning the prevalence of self-management practices among stroke survivors in the United States (U.S.). Even though existing studies have estimated the prevalence of different risk factors among stroke survivors separately, a consolidated prevalence estimate of multiple risk factors is lacking [5,6,7]. Furthermore, disparities in sociodemographic factors such as race have influenced stroke care [8,9]. To address these disparities, recent studies have aimed at targeted interventions among minority stroke survivors [10,11,12,13,14,15,16]. Whether sociodemographic factors such as race, age, income, education, and access to healthcare plans influence self-management is an open problem that has not been comprehensively addressed yet. In a recent cross-sectional study, education level was one of the key predictors affecting self-management among stroke survivors in Taiwan [17]. However, to the best of our knowledge, there are no studies exploring the link between sociodemographics and self-management among stroke survivors in the United States. Understanding the extent to which stroke survivors engage in self-management is crucial for optimizing post-stroke care and improving long-term outcomes. Furthermore, an exploration of the factors that influence self-management strategies among stroke survivors is essential. Factors such as demographics, access to healthcare plans, income, and geography may contribute to the variability in self-management practices.

This study aims to estimate self-management prevalence among stroke survivors in the United States between 2016 and 2021. We further analyze each self-management behavior to determine its prevalence and associated factors. We hypothesized that sociodemographic and geographic factors will impact self-management practices.

## 2. Methods

### 2.1. Design

In this study, we processed and analyzed data from the Behavioral Risk Factor Surveillance System (BRFSS) administered by the Centers for Disease Control and Prevention (CDC) from 2016 to 2021 [18,19,20,21,22,23]. The BRFSS is an annual, cross-sectional telephone survey of household adults residing in the United States (U.S.) and its territories. The BRFSS gathers respondents’ information about diseases, risk factors, self-care practices, and health-related behaviors. BRFSS randomly selects telephone numbers, and calls are made seven days a week throughout the day to avoid selection bias [18]. The response rate in BRFSS in the most recent year (2021) is 44%, and BRFSS provides sample weights to account for nonresponse rates while ensuring the resulting weights represent the U.S. population. We adhered to the STROBE guidelines to report our findings [24].

### 2.2. Eligibility Criteria

The BRFSS survey included household adults 18 years of age or older. A total of 2,632,673 records were initially considered for analysis, out of which 2,522,240 non-stroke survivors were excluded. Of the remaining 110,433 records, 14,779 (13%) had at least one of the data missing for the self-management questions. After eliminating the 14,779 records, a complete analysis was performed on 95,645 records (as shown in Figure S1 in the Supplementary Materials). Respondents with stroke were identified based on self-reports. Evidence suggests the validity and reliability of self-reported data gathered in BRFSS [25]. Prevalence estimates for common conditions such as stroke closely match those derived from in-person examinations, showing a maximum absolute variance of 0.8% between the two datasets [26].

### 2.3. Variable Definitions

Demographic and socioeconomic variables were defined based on responses. Missing values and “do not know/refused” were excluded. Race variables included White only (non-Hispanic), Black only (non-Hispanic), other races only (non-Hispanic), multiracial (non-Hispanic), and Hispanic. Age was categorized as 18 to 64 or 65 and older. Respondents were either classified as with or without medical insurance. Education levels were defined as “did not graduate high school, graduated high school, attending college or technical school, and graduated from college or technical school.” Income levels were defined as less than $15,000, $15,000 to less than $25,000, $25,000 to less than $35,000, $35,000 to less than $50,000, and $50,000 or more. The respondent’s sex was either male or female (Table S1 includes the list of variables along with BRFSS codes).

### 2.4. Outcome Measures

The American Heart Association/American Stroke Association (AHA/ASA) recommends several self-care and self-management practices to prevent, delay, and effectively manage stroke, including strategies ranging from smoking cessation to regular doctor checkups [27]. We identified five specific questions from the BRFSS questionnaire that consistently align with these recommendations between 2016 and 2021. The first two questions were direct inquiries for which we analyzed the responses. These questions encompass the following: “How long has it been since you last visited a doctor for a routine checkup?” and “During the past month, other than your regular job, did you participate in any physical activities or exercises such as running, calisthenics, golf, gardening, or walking for exercise?” The last three questions involve calculated variables within the BRFSS data, from which we extracted values. These calculated variables are related to respondents’ smoking status (current, former, or never smokers), body mass index (underweight, normal weight, overweight, obese), and those who reported consuming more than 14 drinks per week for men or seven drinks per week for women (Table S2 includes inquiries along with BRFSS variables).

We defined a new binary outcome variable called stroke self-management (SSM; SSM = low or SSM = high) based on the following five criteria: (1) presenting for a routine medical checkup within the past year, (2) engaging in physical activities or exercises in the last 30 days, (3) former or never smokers, (4) maintaining normal weight, and (5) consuming less than 14 drinks (for males) or seven drinks (for females) per week. Each respondent scored one or zero based on satisfying the above conditions. The total accumulated score was calculated for each respondent. Given the score distribution, score 3 being the median (Figure S2), we considered a score between 0–3 as ‘low’ and 4–5 as ‘high’.

### 2.5. Statistical Analysis

We merged data from the 2016 to 2021 BRFSS annual surveys into a pooled data set. To calculate population-weighted frequencies and proportions, we used survey-specific R functions to account for survey design, weights, and strata. We used the Rao–Scott chi-squared test to assess differences in ‘low’ SSM percentages across strata (e.g., male vs. female).

We report the sociodemographic characteristics of the population and the percentage of respondents whose SSM is low for all the demographic variables. We conducted single-variate (unadjusted) and multi-variate (adjusted) regression analyses to measure the associations of sociodemographic and demographic factors with the defined SSM variable. For each of the factors, unadjusted and adjusted (adjusted with age, sex, race, education, insurance, income, as well as comorbidities: myocardial infarction, angina or coronary heart disease, asthma, depression, and chronic obstructive pulmonary disease), the odds ratio was calculated. Subsequently, we analyzed the associations of sociodemographic factors on each self-management behavior following the same analytic method stated above. Lastly, we analyzed the distribution of ‘low’ SSM in stroke survivors in the U.S. geographically and followed it up with subgroup analysis by stratifying for each race. Statistical analysis was performed in R 4.0.5. State-wise ‘low’ SSM percentages were plotted using Data Wrapper. Forest plots for odds ratios were generated using the ggplot package in R. *p* < 0.05 was considered statistically significant.

## 3. Results

### 3.1. General Characteristics of the Studied Population

We analyzed BRFSS data. The BRFSS survey included adults 18 years or older. Among the stroke survivors, 51.1% percent were women, and 51.2% were 65 or older—66.4% identified as White only, 16.4% as Black only, and 10.6% as Hispanic. Approximately 26.3% resided in the Stroke Belt (Alabama, Arkansas, Georgia, Kentucky, Louisiana, Mississippi, North Carolina, South Carolina, Tennessee, and Virginia), and 7% lacked health insurance. A total of 21.6% did not graduate from high school, 30.9% graduated from high school, 30.8% attended college or technical school, and 16.6% graduated college or technical school. Table 1 includes the general characteristics of the study population.

### 3.2. Prevalence of Self-Management among Stroke Survivors in the United States

Of the overall population, 46.7% of stroke survivors’ SSM was ‘low’ (0–3 self-management conditions met), and 53.3% SSM was ‘high’ (4–5 self-management conditions fulfilled). Of the five self-management conditions, body mass index (BMI) and physical activity were the least met conditions with 26.6% and 58.7% of the stroke survivors meeting these conditions, respectively, whereas alcohol consumption (96%) and getting annual checkups (90%) were the most met conditions (Table S4). ‘Low’ SSM percentages varied across sociodemographic factors (Table 2). Younger stroke survivors (18 to 64 years) were less likely to self-manage than stroke survivors who are 65 or older (56.8% vs. 42.3%; *p* < 0.0001). Variation with race and ethnicity was observed, with Hispanic and non-Hispanic Blacks being less likely to self-manage when compared to non-Hispanic Whites (52.0% versus 48.6%; *p* < 0.0001). Education played a significant role in self-management; stroke survivors with higher education levels were more prone to self-manage (‘low’ SSM percentage of 32.5% for college graduates versus 60.9% for stroke survivors who did not graduate high school; *p* < 0.0001).

### 3.3. Association of Sociographic and Demographic Factors with SSM and Each of the Self-Management Conditions

Correlation of sociographic and demographic factors with SSM: Stroke survivors 65 or older are more likely to self-manage than younger (less than 65 years) stroke adults (adjusted odds ratio, 1.7 [95% CI, 1.56–1.86] (Table 3). Stroke survivors with higher education (graduated from college or technical school) and income levels ($50,000 or more) are much more likely (adjusted odds ratio, 2.49 [95% CI, 2.16–2.88] and adjusted odds ratio, 1.45 [95% CI, 1.26–1.87], respectively) to self-manage than stroke survivors with lower education (did not graduate high school) and income levels (less than $15,000). Stroke survivors with no insurance are less likely to self-manage than survivors with insurance (odds ratio, 0.46 [95% CI, 0.38–0.56]). The unadjusted odds ratio of stroke self-management behaviors among Blacks indicated they were less likely to self-manage compared to Whites, but after accounting for age, sex, education, insurance, income, and comorbidities, no significant results were obtained.

**Figure 1:**
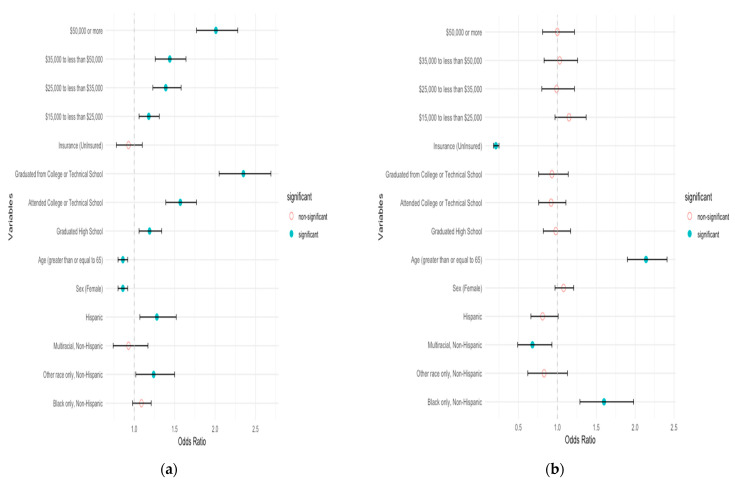

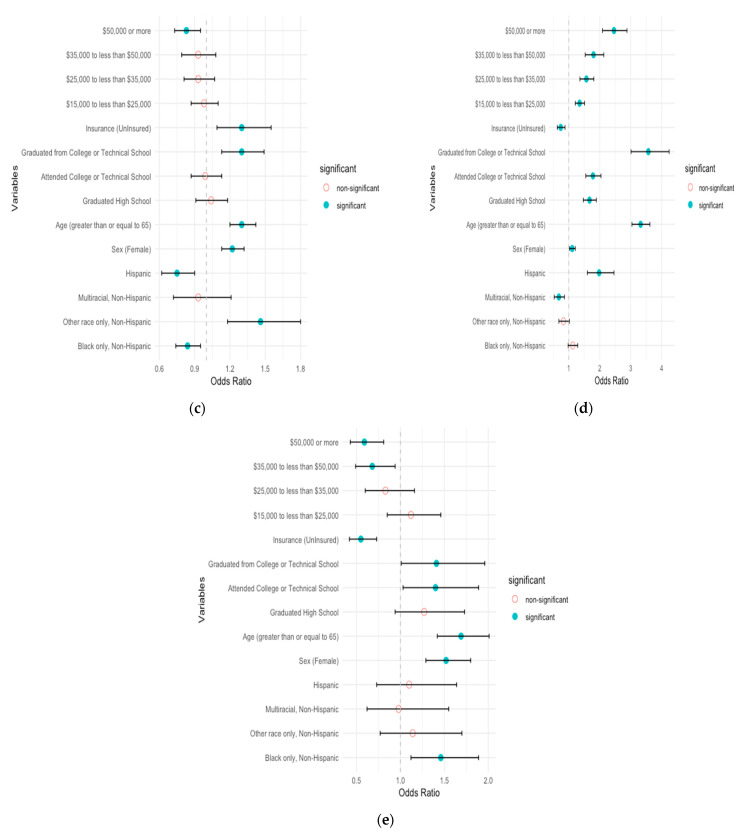
Adjusted odds ratio of self-management among stroke survivors in the United States. (**a**) Physical activity; (**b**) annual checkup; (**c**) BMI; (**d**) smoking; (**e**) alcohol.

Correlation of sociographic and demographic factors with each of the self-management conditions: Female stroke survivors are less likely to be physically active than male stroke survivors (adjusted odds ratio, 0.88 [0.81–0.95]). See Figure 1a and Table S5. Stroke survivors aged 65 and above are less likely to be physically active than younger stroke adults. Stroke survivors with higher education (graduated from college or technical school) and income levels ($50,000 or more) are much more likely (adjusted odds ratio, 2.25 [95% CI, 1.94–2.61] and 1.65 [95% CI, 1.44–1.9], respectively) to be physically active. Examining adherence to regular checkup guidelines, Black stroke survivors are more likely to undergo annual checkups (adjusted odds ratio, 1.77 [95% CI, 1.42–2.19]) (see Figure 1b and Table S6). Older survivors (65 or above) adhere to regular annual checkups (adjusted odds ratio, 2.04 [95% CI, 1.78–2.34]), while uninsured individuals are less likely (adjusted odds ratio, 0.2 [95% CI, 0.17–0.25]).

In BMI management, Black and Hispanic survivors are less likely to maintain healthy BMI (adjusted odds ratio, 0.86 [95% CI, 0.75–0.99] and 0.69 [95% CI, 0.56–0.86], respectively) (see Figure 1c and Table S7). Females, older survivors (65 or above), and those with college degrees are more likely to self-manage BMI (adjusted odds ratio, 1.27 [95% CI, 1.16–1.4], 1.35 [95% CI, 1.23–1.49], and 1.3 [95% CI, 1.13–1.49], respectively).

In smoking self-management correlations, Hispanic survivors show a lower likelihood of smoking compared to White survivors (adjusted odds ratio, 2.06 [95% CI, 1.63–2.62]) (see Figure 1d and Table S8). Additionally, females and older survivors (65 or above) exhibit a higher likelihood of not smoking (adjusted odds ratio, 1.21 [95% CI, 1.09–1.34] and 3.34 [95% CI, 3.02–3.7], respectively). Stroke survivors with higher education and income levels are more likely not to smoke (adjusted odds ratio, 3.31 [95% CI, 2.74–4] and 2.09 [95% CI, 1.74–2.5], respectively), while individuals without insurance are more likely to smoke (adjusted odds ratio, 0.74 [95% CI, 0.61–0.89]).

For alcohol consumption management, Black survivors are more likely to self-manage compared to White survivors (adjusted odds ratio, 1.44 [95% CI, 1.07–1.94]) (see Figure 1e and Table S9). Females and older survivors (65 or above) also exhibit a higher likelihood of managing alcohol consumption (adjusted odds ratio, 1.57 [95% CI, 1.29–1.92] and 1.66 [95% CI, 1.31–2.09], respectively). Conversely, being uninsured and having higher income levels are both negatively associated with self-management of alcohol consumption (adjusted odds ratio, 0.57 [95% CI, 0.41–0.79] and 0.54 [95% CI, 0.36–0.8], respectively).

### 3.4. Prevalence of Self-Management Geographically

**Figure 2:**
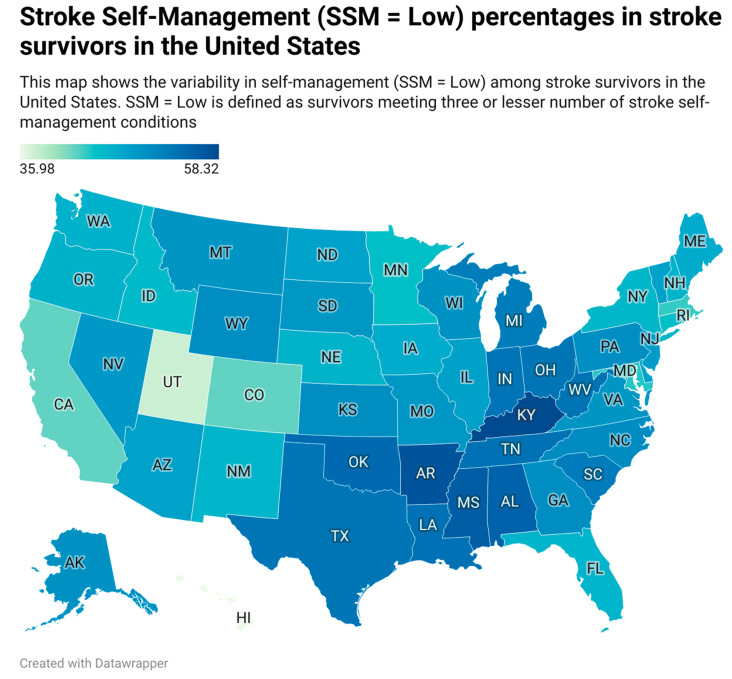
SSM percentages in stroke survivors in the United States. AL: Alabama, AK: Alaska, AZ: Arizona, AR: Arkansas, CA: California, CO: Colorado, CT: Connecticut, DE: Delaware, DC: District of Columbia, FL: Florida, GA: Georgia, HI: Hawaii, ID: Idaho, IL: Illinois, IN: Indiana, IA: Iowa, KS: Kansas, KY: Kentucky, LA: Louisiana, ME: Maine, MD: Maryland, MA: Massachusetts, MI: Michigan, MN: Minnesota, MS: Mississippi, MO: Missouri, MT: Montana, NE: Nebraska, NV: Nevada, NH: New Hampshire, NJ: New Jersey, NM: New Mexico, NY: New York, NC: North Carolina, ND: North Dakota, OH: Ohio, OK: Oklahoma, OR: Oregon, PA: Pennsylvania, RI: Rhode Island, SC: South Carolina, SD: South Dakota, TN: Tennessee, TX: Texas. UT: Utah. VT: Vermont, VA: Virginia, WA: Washington, WV: West Virginia, WI: Wisconsin, WY: Wyoming.

Considerable variability in self-management was noted geographically (Figure 2). Firstly, the ‘low’ SSM percentage varied between states, ranging from 35.9% in Hawaii to 58.3% in Kentucky. Secondly, stroke survivors residing in the Stroke Belt did not self-manage as well as other regions (53.1% vs. 48.0%; *p* < 0.001). Lastly, non-rural residents self-managed better than rural residents (49.1% vs. 52.2%; *p* < 0.05). We also analyzed how self-management varied across states for different races (Figures S3–S7). Whites in the Stroke Belt showed a poorer SSM than other regions. Blacks and Hispanics in North Dakota, South Dakota, and Vermont have a higher percentage of ‘low’ SSM.

## 4. Discussion

In our analysis of nationally representative U.S. BRFSS data spanning from 2016 to 2021, we estimated that the low self-management rate among stroke survivors is 46%. Approximately one in every two stroke survivors manages either three or fewer risk factors. Self-management among stroke survivors varied across sociographic and demographic factors. Education, income, insurance, and age showed positive associations with self-management. A unique set of associations emerged when specific self-management behaviors were analyzed individually. Women and older stroke survivors (65 and above) are physically inactive when compared to their counterparts. Stroke survivors under 65 are less likely to undergo regular checkups. However, females with higher education and income and individuals of the Black race demonstrate better adherence to regular checkups. Females and 65-or-older stroke survivors are more likely to maintain their BMI, whereas individuals belonging to Black and Hispanic races have a lesser likelihood of maintaining their BMI. Stroke survivors with higher income levels are better at adhering to smoking guidelines but worse at managing their alcohol consumption. Self-management varied geographically, with residents in the Stroke Belt not self-managing as well as their counterparts.

Our analysis provides a benchmark on self-management based on large, nationally representative survey data. To the best of our knowledge, this study is the first exploration of its kind; there is no basis for comparison with the results of any existing studies. However, the prevalence of some of the self-management conditions was assessed individually in previous studies [5,6,7]. Our results are consistent concerning the prevalence of behaviors such as alcohol consumption, smoking, and BMI in stroke survivors. However, these studies report their findings based on data from a single year of BRFSS survey and do not explore the impact of sociographic and demographic factors.

Our findings regarding older stroke survivors and women being physically inactive are consistent with similar prior studies on 2003 BRFFS data [28]. Our findings revealed that younger stroke survivors (<65 years old) do not manage their risk factors as well as older stroke survivors, and a significant gap exists between the two groups. Additionally, we found that education and income levels are important in how well stroke survivors manage their risk factors. These findings may help inform the development of stroke interventions for specific target populations.

Furthermore, our results show that physical activity and weight management are two behaviors that are ill-managed among stroke survivors. This finding is not only consistent with the current literature but the literature also suggests that stroke survivors are physically inactive compared to survivors of other chronic diseases [29], Although physical activity is the most effective modifiable behavior for the prevention of recurrent stroke [30], stroke survivors have difficulty maintaining the recommended levels of physical activity. There is a vital need for novel, contextualized, and individualized physical activity interventions (physical therapy, health coaching, or group sessions) designed for stroke survivors to fill this gap.

Our findings revealed stroke survivors with a college degree or higher are 2.5 times more likely to self-manage than stroke survivors without a high school degree. This result emphasizes the importance of education in self-management [31] and is further solidified by the evidence that effective self-management interventions have education as a vital intervention component [32,33,34]. This result reinforces the necessity of interventions aimed at improving patient education about stroke.

Based on our findings, younger, less educated, and lower-income stroke survivors are less likely to self-manage than their counterparts. We recommend that newer health policies focus on these specific targeted populations to improve the prevention of stroke recurrence. We further recommend randomized control trials to objectively validate the results obtained from this study.

This study boasts significant strengths, primarily attributed to our utilization of recent extensive, nationally represented health survey data spanning seven years and our empirical categorization of low and high self-management. However, our study also has limitations: (1) Self-management is a multi-dimensional problem, and because of the lack of data on multiple events, the results do not reflect the confounding effects of various conditions. Pre-morbid disability, stroke severity, and physical activity levels pre-stroke may all influence stroke survivors’ self-management. Additionally, stroke survivors’ access to caretakers and nurses also influences their self-management, which is not accounted for in this study. (2) BRFSS is a cross-sectional study; therefore, it cannot be used to establish cause–effect relationships. However, being a cross-sectional study, it is best suited to estimate prevalence, which is the primary objective of this study. (3) Respondents to the BRFSS survey are those who volunteered, and this dataset is missing institutionalized stroke survivors. However, BRFSS data is weighted for noncoverage and nonresponse. (4) Respondents may have modestly overestimated their management of certain risk factors, such as physical activity, whereas they might have underestimated factors, such as obesity and being overweight [35]. This bias is not accounted for in our analysis. (5) Although BMI was one of the self-management practices analyzed, because of the limitations of the dataset, our findings do not account for the dietary habits of stroke survivors, which directly influence BMI. (6) BRFSS does not distinguish ischemic and hemorrhagic stroke survivors. About 87% of strokes are ischemic [36]; hence, the prevalence results of self-management in this study can be generalized. (7) Access to healthcare resources and cost-effectiveness of self-management practices play a significant role in how a stroke survivor self-manages; however, these factors are not taken into account because of the lack of data. (8) Self-management encompasses a wide range of practices and is influenced by varied comorbidities and environmental factors. The BRFSS survey only accommodates a subset of these practices and comorbidities, which are taken into account in this analysis.

## 5. Conclusions

The substantial diversity in self-management practices among stroke survivors emphasizes the need for tailored interventions. In particular, multi-modal interventions should be tailored for specific populations and regions, addressing the needs of stroke survivors under 65 with lower educational attainment and income levels, along with implementing interventions for residents in Stroke Belt and rural areas. When targeting a particular behavior, a more precise approach is recommended, such as promoting physical activity among Black female stroke survivors aged 65 or older, or educating younger stroke survivors (less than 65 years) to get annual doctor checkups. Recognizing these variations allows for more effective and equitable post-stroke care interventions, ensuring improved outcomes for diverse populations.

## Figures and Tables

**Table 1:** General Characteristics of the Studied Population.

Factor	Whole Population (Weighted) %
Respondents, raw unweighted frequency	95,645
Race	
White only, non-Hispanic	66.4
Black only, non-Hispanic	16.4
Other races only, non-Hispanic	4.6
Multiracial, non-Hispanic	1.8
Hispanic	10.6
Sex	
Male	48.8
Female	51.1
Age	
18 to 64	48.7
65 or older	51.2
Education	
Did not graduate high school	21.6
Graduated high school	30.9
Attended college or technical school	30.8
Graduated from college or technical school	16.6
Lacking health insurance	7.0
Rural residence	22.9
Stroke Belt residence	26.3
Income	
Less than $15,000	20.5
$15,000 to less than $25,000	25.7
$25,000 to less than $35,000	12.9
$35,000 to less than $50,000	13.1
$50,000 or more	27.6

**Table 2:** Demographic factors and SSM percentage (SSM = low) among stroke survivors.

Factor	N (Unweighted)	Low SSM Percentage (95% CI) (Weighted)	*p*-Value
Age			*p* < 0.0001
18 to 64 years old	35,125	56.8 (55.7–57.9)	
65 or older	59,865	42.3 (41.4–43.2)	
Sex			*p* = 0.6
Male	43,445	49.2 (48.1–50.2)	
Female	52,170	49.5 (48.5–50.5)	
Race and ethnicity			*p* < 0.0001
White only, non-Hispanic	71,880	48.6 (47.8–49.4)	
Black only, non-Hispanic	10,351	52.0 (50.0–53.9)	
Other races only, non-Hispanic	4467	43.4 (39.1–47.6)	
Multiracial, non-Hispanic	2506	53.3 (48.6–58.0)	
Hispanic	4610	52.0 (48.7–55.4)	
Health insurance			*p* < 0.0001
Insured	76,730	47.8 (47.0–48.7)	
Uninsured	3892	69.6 (66.4–72.9)	
Stroke Belt			*p* < 0.0001
Stroke Belt residents	21,334	53.1 (52.1–54.2)	
Non-Stroke Belt residents	74,311	48.0 (47.1–48.9)	
Rurality			*p* < 0.05
Nonrural	17,519	49.1 (48.3–49.9)	
Rural	30,787	52.2 (50.2–54.1)	
Education			*p* < 0.0001
Did not graduate high school	11,779	60.9 (59.0–62.7)	
Graduated high school	30,978	53.0 (51.8–54.2)	
Attended college or technical school	28,237	46.8 (45.5–48.1)	
Graduated from college or technical school	24,423	32.5 (31.2–33.8)	
Income			*p* < 0.0001
Less than $15,000	15,223	62.5 (60.7–64.3)	
$15,000 to less than $25,000	20,169	54.1 (52.5–55.6)	
$25,000 to less than $35,000	10,808	49.5 (47.4–51.6)	
$35,000 to less than $50,000	10,827	45.1 (42.9–47.3)	
$50,000 or more	22,005	39.1 (37.6–40.6)	

**Table 3:** Odds ratio of stroke self-management in the United States. Adjusted for non-modifiable and modifiable factors (sex, race, age, education, insurance, income, myocardial infarction, angina or coronary heart disease, asthma, depression, chronic obstructive pulmonary disease).

Variables (Reference)	Odds Ratio Unadjusted (95% CI)	*p*-Value	Odds Ratio Adjusted (95% CI)	*p*-Value
Race	-	-	-	-
White (reference)				
Black only, non-Hispanic	0.87 (0.8–0.95)	0.0016	1.04 (0.92–1.17)	0.5546
Other races only, non-Hispanic (comes at the end)	1.23 (1.04–1.47)	0.0187	1.27 (1.01–1.59)	0.0414
Multiracial, non-Hispanic	0.83 (0.68–1)	0.0556	0.9 (0.72–1.13)	0.3788
Hispanic	0.87 (0.76–1)	0.0468	1.2 (0.99–1.45)	0.0689
Sex				
Male (reference)	-	-	-	-
Female	0.98 (0.93–1.04)	0.6071	1.07 (0.99–1.16)	0.092
Age				
Less than 65 (reference)	-	-	-	-
65 and above	1.8 (1.69–1.91)	<0.0001	1.70 (1.56–1.86)	<0.0001
Education				
Did not graduate high school (reference)	-	-	-	-
Graduated high school	1.38 (1.26–1.51)	<0.0001	1.31 (1.15–1.49)	<0.0001
Attended college or technical school	1.77 (1.61–1.94)	<0.0001	1.71 (1.5–1.94)	<0.0001
Graduated from college or technical school	3.23 (2.93–3.57)	<0.0001	2.49 (2.16–2.88)	<0.0001
Insurance				
Insured (reference)	-	-	-	-
Uninsured	0.4 (0.34–0.47)	<0.0001	0.46 (0.38–0.56)	<0.0001
Income				
Less than $15,000 (reference)	-	-	-	-
$15,000 to less than $25,000	1.41 (1.28–1.56)	<0.0001	1.14 (1.01–1.28)	<0.05
$25,000 to less than $35,000	1.7 (1.52–1.9)	<0.0001	1.21 (1.05–1.4)	<0.05
$35,000 to less than $50,000	2.03 (1.8–2.28)	<0.0001	1.3 (1.13–1.51)	<0.0001
$50,000 or more	2.6 (2.35–2.87)	<0.0001	1.45 (1.26–1.67)	<0.0001

## Data Availability

The data presented in this study are available at CDC—BRFSS Annual Survey Data.

## Supplementary Materials

The following supporting information can be downloaded at https://www.mdpi.com/article/10.3390/jcm13154338/s1: Figure S1: Inclusion/exclusion criteria for the study; Figure S2: Self-management score distribution; Figure S3: SSM percentages in stroke survivors of the White race; Figure S4: SSM percentages in stroke survivors of the Black race; Figure S5: SSM percentages in stroke survivors of other races; Figure S6: SSM percentages in multiracial stroke survivors; Figure S7: SSM percentages in stroke survivors of the Hispanic race; Table S1: Final variables used in the study based on BRFSS codebook; Table S2: Questions in BRFSS for determining self-management variable stroke self-management (SSM); Table S3: Unweighted count supporting Table 1; Table S4: Sociodemographic characteristics for each of the 5 self-management categories with 95% confidence intervals, representing the percentage of the population which meets each of the self-management condition; Table S5: Odds ratio of physical activity in the United States; Table S6: Odds ratio of checkups in the United States; Table S7: Odds ratio of BMI in the United States; Table S8: Odds ratio of smoking in the United States; Table S9: Odds ratio of alcohol consumption in the United States.
