# Supplementary material for: Self-Management among Stroke Survivors in the United States, 2016 to 2021
Source: J Clin Med. 2024 Jul 25;13(15):4338. doi: 10.3390/jcm13154338 (PMC11313262; doi:10.3390/jcm13154338)
Supplement: Supplementary file 1 [file jcm-13-04338-s001.zip › jcm-3092139-supplementary.pdf]

Self-Management among Stroke Survivors in the United States, 2016 to 2021

Figure S1. Inclusion/Exclusion criteria for the study

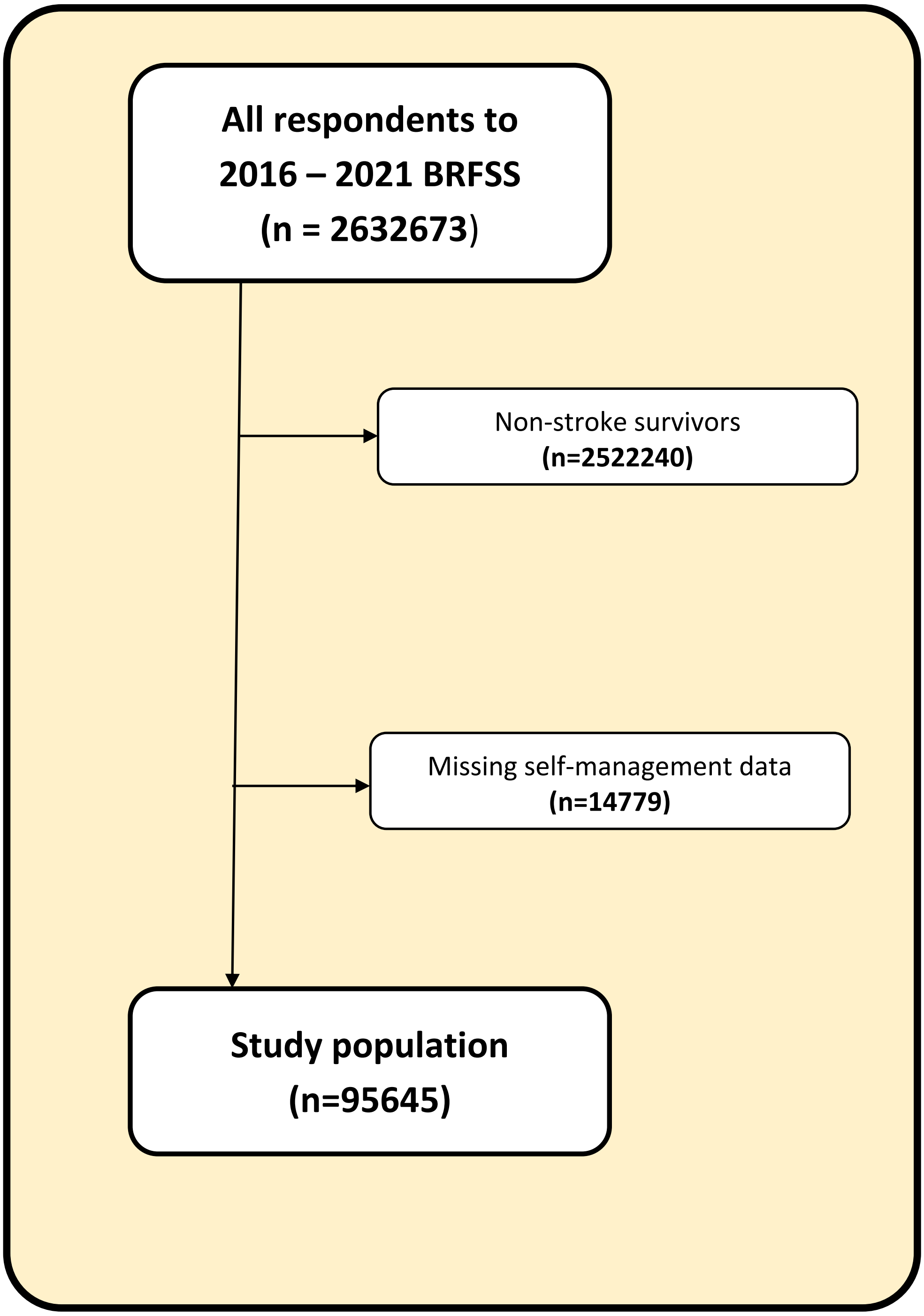

Table S1. Final variables used in the study based on BRFSS codebook

Variable	Variable Name in BRFSS codebook	Variable Categories in BRFSS codebook	Re-Coded Values
Age	**_AGE65YR**	18 to 64	1
		65 or older	2
SEX	**SEX, _SEX, SEX1**	Male	1
		Female	2
RACE	**_RACEGR3**	White only, Non-Hispanic	1
		Black only, Non-Hispanic	2
		Other race only, Non-Hispanic	3
		Multiracial, Non-Hispanic	4
		Hispanic	5
Education	**_EDUCAG**	Did not graduate High School	1
		Graduated High School	2
		Attended College or Technical School	3
		Graduated from College or Technical School	4
Insurance	**HLTHPLN1**	Yes	1
		No	2
Metropolitan	**MSCODE**	In the center city of a Metropolitan Status Area (MSA)	Urban
		Outside the center city of an MSA but inside the county containing the center city	Urban
		Inside a suburban country of the MSA	Urban
		Not in an MSA	Rural
Stroke belt residence	**STRKBLT Derived variable for this study.**	Alabama, Arkansas, Georgia, Louisiana, Mississippi, North	Yes

		Carolina, South Carolina, and Tennessee	
		Other States	No
Stroke Survivor	**CVDSTRK3**	Yes	1
		No	2

Table S2. Questions in BRFSS for determining self-management variable Stroke Self-Management (SSM)

**BRFSS Question**	**BRFSS Variable**	**Variable Categories in BRFSS**	**Re-Coded Values**
About how long has it been since you last visited a doctor for a routine checkup?	**CHECKUP1**	Within past year (anytime less than 12 months ago)	1
		Within past 2 years (1 year but less than 2 years ago)	2
		Within past 5 years (2 years but less than 5 years)	3
		5 or more years ago	4
Four-level smoker status (calculated variable by BRFSS)	**_SMOKER3**	Current smoker – now smokes every day	Smoker
		Current smoker – now smokes some days	Smoker
		Former smoker	Non-smoker
		Never smoked	Non-smoker
Heavy drinkers (adult men having more than 14 drinks per week and adult women having more than 7 drinks per week)	**_RFDRHV5, _RFDRHV6, _RFDRHV7, _RFDRHV8, _RFDRHV9 (different variable names for different years)**	No	2
		Yes	1
Adults who reported doing physical activity or exercise during the past 30 days other than their regular job	**_TOTINDA**	Had physical activity or exercise	1
		No physical activity or exercise in last 30 days	2
	**_BMI5CAT**	Underweight	1

Four categories of Body Mass Index (BMI) (calculated variable by BRFSS)		Normal Weight	2
		Overweight	3
		Obese	4

Figure S2: SSM score distribution

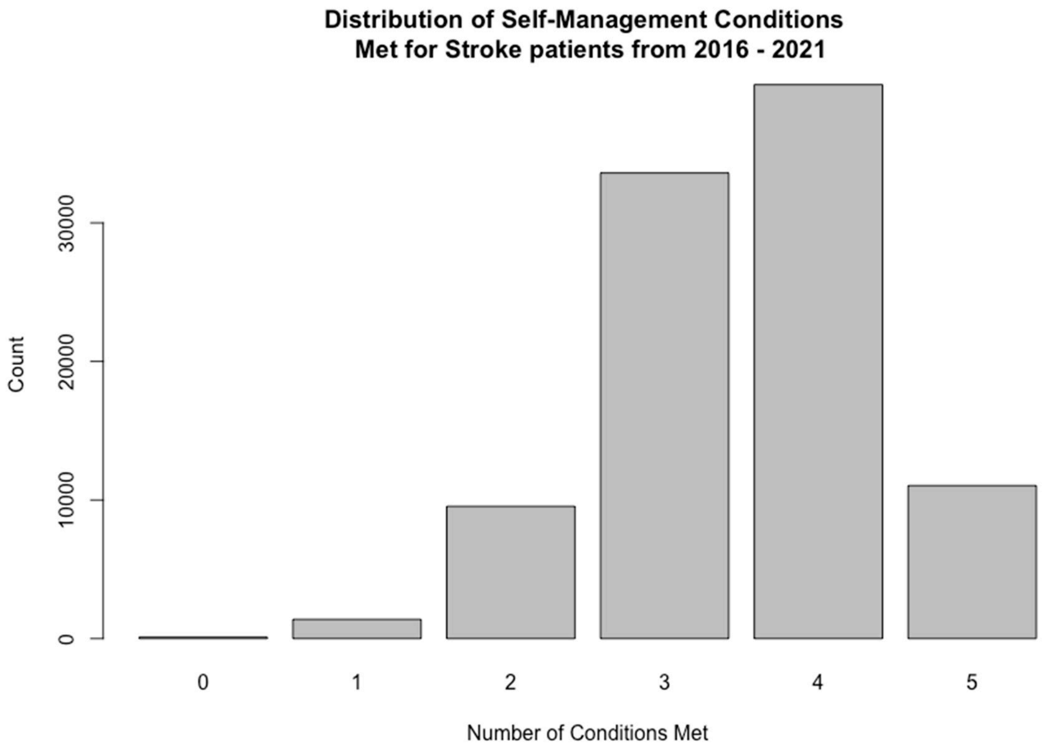

Table S3. Unweighted count supporting Table 1

**Factor**	**Unweighted Frequency of respondents % (CI95%)**
Respondents, raw unweighted frequency	95645
Respondents, U.S. weighted frequency	
**Race**	
White only, Non Hispanic	71880
Black only, Non-Hispanic	10351
Other race only, Non-Hispanic	4467
Multiracial, Non-Hispanic	2506
Hispanic	4610

**SEX**	
Male	43445
Female	52170
**Age**	
18 to 64	35125
65 or older	59865
**Education**	
Did not graduate High School	11779
Graduated High School	30978
Attended College or Technical School	28237
Graduated from College or Technical School	24423
**Lacking Health Insurance**	3892
**Rural residence**	17519
**Stroke belt**	21334

Table S4. Sociodemographic characteristics on each of the 5 Self Management categories with 95% confidence Intervals. Represents the percentage of the population which meets each of the Self Management condition

**SocioDemographic Factor**	**Checkup**	**BMI**	**Physical Activity**	**Smoking**	**Alcohol**
**Age**					
18 to 64	84.3 (83.5 – 85.1)	22.6 (21.6 – 23.5)	59.4 (58.3 – 60.5)	66.8 (65.7 – 67.8)	94.4 (93.9 – 95.0)
65 or older	93.5 (93.1 – 94.0)	28.1 (27.3 – 28.9)	57.1 (56.1 – 58.0)	87.5 (87.0 – 88.1)	96.9 (96.6 – 97.2)
**Sex**					
Male	88.1 (87.4 – 88.7)	23.4 (22.6 – 24.3)	61.0 (59.9 – 62.2)	76.8 (75.9 – 77.7)	94.5 (94.1 – 95.1)
Female	89.9 (89.2 – 90.5)	27.4 (26.5 – 28.3)	55.7 (54.7 – 56.7)	78.0 (77.2 – 78.9)	96.8 (96.4 – 97.1)
**Race and Ethnicity**					
White only, Non-Hispanic	89.3 (88.8 – 89.8)	26.4 (25.7 – 27.0)	58.5 (57.7 – 59.2)	78.0 (77.3 – 78.6)	95.4 (95.0 – 95.8)

Black only, Non-Hispanic	91.9 (90.8 – 93.0)	22.4 (20.8 – 24.1)	56.6 (54.7 – 58.6)	74.5 (72.6 – 76.4)	96.9 (96.3 – 97.5)
Other race only, Non-Hispanic	87.2 (84.7 – 89.8)	35.9 (31.3 – 40.4)	62.1 (57.8 – 66.4)	75.0 (71.7 – 78.4)	95.7 (94.5 – 97.0)
Multiracial, Non-Hispanic	83.2 (79.5 – 86.9)	26.7 (21.8 – 31.6)	58.1 (53.5 – 62.7)	66.7 (62.2 – 71.2)	96.1 (94.6 – 97.6)
Hispanic	84.7 (82.5 – 86.8)	20.1 (17.8 – 22.5)	57.1 (53.8 – 60.4)	81.8 (79.5 – 84.1)	95.8 (94.6 – 97.1)
**Health Insurance**					
Insured	90.8 (90.4 – 91.3)	25.3 (24.6 – 26.0)	58.4 (57.6 – 59.2)	78.5 (77.8 – 79.2)	96.0 (95.6 – 96.3)
Uninsured	64.2 (61.0 – 67.5)	28.0 (24.8 – 31.2)	54.4 (50.9 – 57.9)	61.1 (57.5 – 64.7)	91.9 (90.2 – 93.6)
**Stroke belt**					
Stroke belt Residents	89.7 (89.0 – 90.4)	24.7 (23.8 – 25.6)	53.7 (52.7 – 54.8)	74.4 (73.4 – 75.3)	96.4 (96.0 – 96.7)
Non stroke belt Residents	88.7 (88.2 – 89.3)	25.7 (24.9 – 26.5)	59.9 (45.9 – 59.0)	78.5 (77.8 – 79.3)	95.5 (95.1 – 95.9)
**Rurality**					
Non-Rural	88.7 (88.2 – 89.2)	25.5 (24.8 – 26.2)	58.9 (58.1 – 59.7)	77.3 (76.7 – 78.0)	95.6 (95.3 – 96.0)
Rural	91.9 (91.0 – 92.8)	24.9 (23.5 – 26.4)	51.2 (49.2 – 53.2)	78.9 (77.4 – 80.4)	96.6 (95.9 – 97.4)
**Education**					
Did not graduate High School	88.7 (87.7 – 89.8)	24.6 (23.1 – 26.1)	48.0 (46.1 – 49.9)	66.0 (64.2 – 67.8)	95.3 (94.5 – 96.1)
Graduated High School	88.8 (88.0 – 89.6)	25.0 (24.0 – 26.1)	53.8 (52.6 – 55.0)	76.7 (75.7 – 77.7)	95.9 (95.3 – 96.5)
Attended College or Technical School	88.9 (88.0 – 89.8)	24.3 (23.2 – 25.5)	61.4 (60.1 – 62.6)	79.2 (78.2 – 80.3)	96.0 (95.5 – 96.5)
Graduated from College or Technical School	89.8 (89.0 – 90.6)	29.0 (27.8 – 30.3)	74.4 (73.2 – 75.6)	90.2 (89.3 – 91.0)	95.4 (94.9 – 95.9)

Table S5. Odds Ratio of Physical Activity in the United States. Adjusted for non-modifiable and modifiable factors (Sex, Race, Age, Education, Insurance, Income, Myocardial Infarction, Angina or Coronary Heart Disease, Asthma, Depression, Chronic obstructive pulmonary disease)

**Variables (Reference)**	**Odds ratio unadjusted (95% CI)**	**p-Value**	**Odds ratio adjusted (95% CI)**	**p-Value**
**Race**	-	-	-	-
White (Reference)				
Black only, Non-Hispanic	0.93 (0.85 - 1.01)	0.0801	0.95 (0.85 - 1.07)	0.4349
Other race only, Non-Hispanic	1.16 (0.97 - 1.4)	0.1055	1.14 (0.92 - 1.42)	0.219
Multiracial, Non-Hispanic	0.98 (0.81 - 1.19)	0.8718	0.9 (0.69 - 1.16)	0.4
Hispanic	0.95 (0.82 - 1.09)	0.4287	1.04 (0.85 - 1.27)	0.7033
**Sex**				
Male (Reference)	-	-	-	-
Female	0.8 (0.76 - 0.85)	< 0.001	0.88 (0.81 - 0.95)	< 0.05
**Age**				
less than 65 (Reference)	-	-	-	-
65 and above	0.91 (0.86 - 0.97)	0.002	0.89 (0.81 - 0.97)	<0.05
**Education**				
Did not graduate High School (Reference)	-	-	-	-
Graduated High School	1.26 (1.15 - 1.38)	< 0.001	1.16 (1.02 - 1.32)	0.0232
Attended College or Technical School	1.72 (1.57 - 1.89)	< 0.001	1.6 (1.41 - 1.83)	< 0.001
Graduated from College or Technical School	3.15 (2.86 - 3.48)	< 0.001	2.25 (1.94 - 2.61)	< 0.001
**Insurance**				
Insured (Reference)	-	-	-	-
Uninsured	0.85 (0.73 - 0.98)	0.0255	0.87 (0.72 - 1.05)	0.1391
**Income**				
less than $15,000 (Reference)	-	-	-	-

$15,000 to less than $25,000	1.2 (1.09 - 1.33)	< 0.001	1.07 (0.95 - 1.2)	0.2435
$25,000 to less than $35,000	1.46 (1.3 - 1.63)	< 0.001	1.2 (1.04 - 1.39)	< 0.05
$35,000 to less than $50,000	1.7 (1.51 - 1.91)	< 0.001	1.23 (1.05 - 1.42)	< 0.05
$50,000 or more	2.71 (2.45 - 3)	< 0.001	1.65 (1.44 – 1.9)	< 0.001

Table S6. Odds Ratio of Check Up in the United States. Adjusted for non-modifiable and modifiable factors (Sex, Race, Age, Education, Insurance, Income, Myocardial Infarction, Angina or Coronary Heart Disease, Asthma, Depression, Chronic obstructive pulmonary disease)

**Variables (Reference)**	**Odds ratio unadjusted (95% CI)**	**p-Value**	**Odds ratio adjusted (95% CI)**	**p-Value**
**Race**	-	-	-	-
White (Reference)				
Black only, Non-Hispanic	1.35 (1.16 - 1.58)	< 0.001	1.78 (1.39 – 2.28)	< 0.001
Other race only, Non-Hispanic	0.82 (0.65 - 1.04)	0.0956	1.04 (0.75 - 1.44)	0.8185
Multiracial, Non-Hispanic	0.59 (0.45 - 0.78)	< 0.001	0.72 (0.49 - 1.07)	0.1006
Hispanic	0.66 (0.56 - 0.79)	< 0.001	0.93 (0.73 - 1.18)	0.5626
**Sex**				
Male (Reference)	-	-	-	-
Female	1.2 (1.09 - 1.32)	< 0.001	1.06 (0.93 - 1.21)	0.4064
**Age**				
less than 65 (Reference)	-	-	-	-
65 or older	2.7 (2.45 - 2.96)	< 0.001	2.04 (1.78 - 2.34)	<0.001
**Education**				
Did not graduate High School (Reference)	-	-	-	-
Graduated High School	1.26 (1.15 - 1.38)	< 0.001	0.98 (0.82 - 1.17)	0.7924

Attended College or Technical School	1.72 (1.57 - 1.89)	< 0.001	0.92 (0.76 - 1.11)	0.3857
Graduated from College or Technical School	3.15 (2.86 - 3.48)	< 0.001	0.93 (0.76 - 1.14)	0.4692
**Insurance**				
Insured (Reference)	-	-	-	-
Uninsured	0.18 (0.16 - 0.21)	< 0.001	0.2 (0.17 - 0.25)	< 0.001
**Income**				
less than $15,000 (Reference)	-	-	-	-
$15,000 to less than $25,000	1.32 (1.14 - 1.53)	0.0002	1.06 (0.88 - 1.29)	0.5253
$25,000 to less than $35,000	1.2 (1 - 1.43)	0.0463	0.93 (0.74 - 1.18)	0.5683
$35,000 to less than $50,000	1.32 (1.11 - 1.57)	0.0018	1.05 (0.84 - 1.32)	0.6803
$50,000 or more	1.15 (0.99 - 1.34)	0.0609	0.92 (0.73 - 1.17)	0.5004

Table S7. Odds Ratio of BMI in the United States. Adjusted for non-modifiable and modifiable factors (Sex, Race, Age, Education, Insurance, Income, Myocardial Infarction, Angina or Coronary Heart Disease, Asthma, Depression, Chronic obstructive pulmonary disease)

**Variables (Reference)**	**Odds ratio unadjusted (95% CI)**	**p-Value**	**Odds ratio adjusted (95% CI)**	**p-Value**
**Race**	-	-	-	-
White (Reference)				
Black only, Non-Hispanic	0.81 (0.73 - 0.89)	< 0.001	0.86 (0.75 - 0.99)	< 0.05
Other race only, Non-Hispanic	1.56 (1.28 - 1.91)	< 0.001	1.36 (1.07 - 1.73)	< 0.05
Multiracial, Non-Hispanic	1.02 (0.79 - 1.31)	0.8886	1.02 (0.75 - 1.39)	0.88
Hispanic	0.7 (0.61 - 0.82)	< 0.001	0.69 (0.56 - 0.86)	<0.001
**Sex**				
Male (Reference)	-	-	-	-
Female	1.23 (1.15 - 1.32)	< 0.001	1.27 (1.16 - 1.4)	< 0.001

**Age**				
less than 65 (Reference)	-	-	-	-
65 or older	1.34 (1.25 - 1.43)	< 0.001	1.35 (1.23 - 1.49)	<0.001
**Education**				
Did not graduate High School (Reference)	-	-	-	-
Graduated High School	1.02 (0.93 - 1.13)	0.6727	1.04 (0.91 - 1.18)	0.5692
Attended College or Technical School	0.99 (0.89 - 1.09)	0.7747	0.99 (0.87 - 1.13)	0.8881
Graduated from College or Technical School	1.25 (1.13 - 1.39)	< 0.001	1.3 (1.13 - 1.49)	< 0.001
**Insurance**				
Insured (Reference)	-	-	-	-
Uninsured	1.15 (0.98 - 1.35)	0.0904	1.22 (1 - 1.47)	< 0.05
**Income**				
less than $15,000 (Reference)	-	-	-	-
$15,000 to less than $25,000	1.04 (0.93 - 1.16)	0.4774	0.98 (0.86 - 1.1)	0.7073
$25,000 to less than $35,000	1.05 (0.93 - 1.18)	0.4784	0.88 (0.75 - 1.03)	0.1026
$35,000 to less than $50,000	1.04 (0.91 - 1.19)	0.5448	0.9 (0.62 - 1.08)	< 0.001
$50,000 or more	0.99 (0.89 - 1.1)	0.8072	0.72 (0.62 - 0.84)	< 0.001

Table S8. Odds Ratio of Smoking in the United States. Adjusted for non-modifiable and modifiable factors (Sex, Race, Age, Education, Insurance, Income, Myocardial Infarction, Angina or Coronary Heart Disease, Asthma, Depression, Chronic obstructive pulmonary disease)

**Variables (Reference)**	**Odds ratio unadjusted (95% CI)**	**p-Value**	**Odds ratio adjusted (95% CI)**	**p-Value**
**Race**	-	-	-	-
White (Reference)				
Black only, Non-Hispanic	0.83 (0.74 - 0.92)	< 0.001	1.11 (0.95 - 1.31)	0.18
Other race only, Non-Hispanic	0.85 (0.71 - 1.02)	0.0843	1 (0.79- 1.26)	0.9695
Multiracial, Non-Hispanic	0.57 (0.46 - 0.69)	< 0.001	0.78 (0.6 – 1.03)	0.0808
Hispanic	1.27 (1.08 - 1.49)	< 0.001	2.06 (1.63 – 2.62)	< 0.001
**Sex**				
Male (Reference)	-	-	-	-
Female	1.07 (1 - 1.15)	0.0458	1.21 (1.09 - 1.34)	< 0.001
**Age**				
less than 65 (Reference)	-	-	-	-
65 and above	3.5 (3.26 - 3.75)	< 0.001	3.34 (3.02 - 3.7)	<0.001
**Education**				
Did not graduate High School (Reference)	-	-	-	-
Graduated High School	1.69 (1.54 - 1.87)	< 0.001	1.6 (1.39 - 1.84)	< 0.001
Attended College or Technical School	1.96 (1.77 - 2.18)	< 0.001	1.78 (1.54 - 2.09)	< 0.001
Graduated from College or Technical School	4.73 (4.17 - 5.36)	< 0.001	3.31 (2.74 - 4.0)	< 0.001
**Insurance**				
Insured (Reference)	-	-	-	-
Uninsured	0.43 (0.37 - 0.5)	< 0.001	0.74 (0.61 - 0.89)	< 0.05
**Income**				

less than $15,000 (Reference)	-	-	-	-
$15,000 to less than $25,000	1.57 (1.42 - 1.74)	< 0.001	1.19 (1.04 - 1.35)	< 0.05
$25,000 to less than $35,000	2.04 (1.8 - 2.31)	< 0.001	1.4 (1.18 - 1.67)	< 0.001
$35,000 to less than $50,000	2.37 (2.06 - 2.72)	< 0.001	1.53 (1.27 - 1.84)	< 0.001
$50,000 or more	3.35 (2.95 - 3.8)	< 0.001	2.09 (1.74 - 2.5)	< 0.001

Table S9. Odds Ratio of Alcohol in the United States. Adjusted for non-modifiable and modifiable factors (Sex, Race, Age, Education, Insurance, Income, Myocardial Infarction, Angina or Coronary Heart Disease, Asthma, Depression, Chronic obstructive pulmonary disease)

**Variables (Reference)**	**Odds ratio unadjusted (95% CI)**	**p-Value**	**Odds ratio adjusted (95% CI)**	**p-Value**
**Race**	-	-	-	-
White (Reference)				
Black only, Non-Hispanic	1.53 (1.23 - 1.9)	< 0.001	1.44 (1.07 – 1.94)	0.015
Other race only, Non-Hispanic	1.09 (0.79 - 1.5)	0.6103	1.14 (0.73 - 1.78)	0.5578
Multiracial, Non-Hispanic	1.2 (0.79 - 1.8)	0.3928	1.15 (0.65 – 2.01)	0.6351
Hispanic	1.12 (0.81 - 1.54)	0.4944	1.19 (0.73 - 1.95)	0.4855
**Sex**				
Male (Reference)	-	-	-	-
Female	1.69 (1.47 - 1.96)	< 0.001	1.57 (1.29 - 1.92)	< 0.001
**Age**				
less than 65 (Reference)	-	-	-	-
65 and above	1.86 (1.61 - 2.15)	< 0.001	1.66 (1.31 - 2.09)	<0.001
**Education**				
Did not graduate High School (Reference)	-	-	-	-

Graduated High School	1.15 (0.91 - 1.44)	0.2359	1.27 (0.94 - 1.73)	0.1239
Attended College or Technical School	1.19 (0.96 - 1.48)	0.1128	1.4 (1.03 - 1.89)	0.0305
Graduated from College or Technical School	1.01 (0.82 - 1.25)	0.9071	1.41 (1.01 - 1.96)	0.0415
**Insurance**				
Insured (Reference)	-	-	-	-
Uninsured	0.43 (0.37 - 0.5)	< 0.001	0.57 (0.41 - 0.79)	< 0.001
**Income**				
less than $15,000 (Reference)	-	-	-	-
$15,000 to less than $25,000	1.24 (0.98 - 1.57)	0.068	0.96 (0.71 - 1.31)	0.8117
$25,000 to less than $35,000	1.02 (0.77 - 1.35)	0.8888	0.71 (0.49 - 1.03)	0.06
$35,000 to less than $50,000	0.86 (0.66 - 1.14)	0.297	0.62 (0.43 - 0.91)	< 0.05
$50,000 or more	0.69 (0.55 - 0.86)	0.0011	0.54 (0.36 - 0.8)	< 0.005

Figure S3: SSM percentages in stroke survivors of White Race

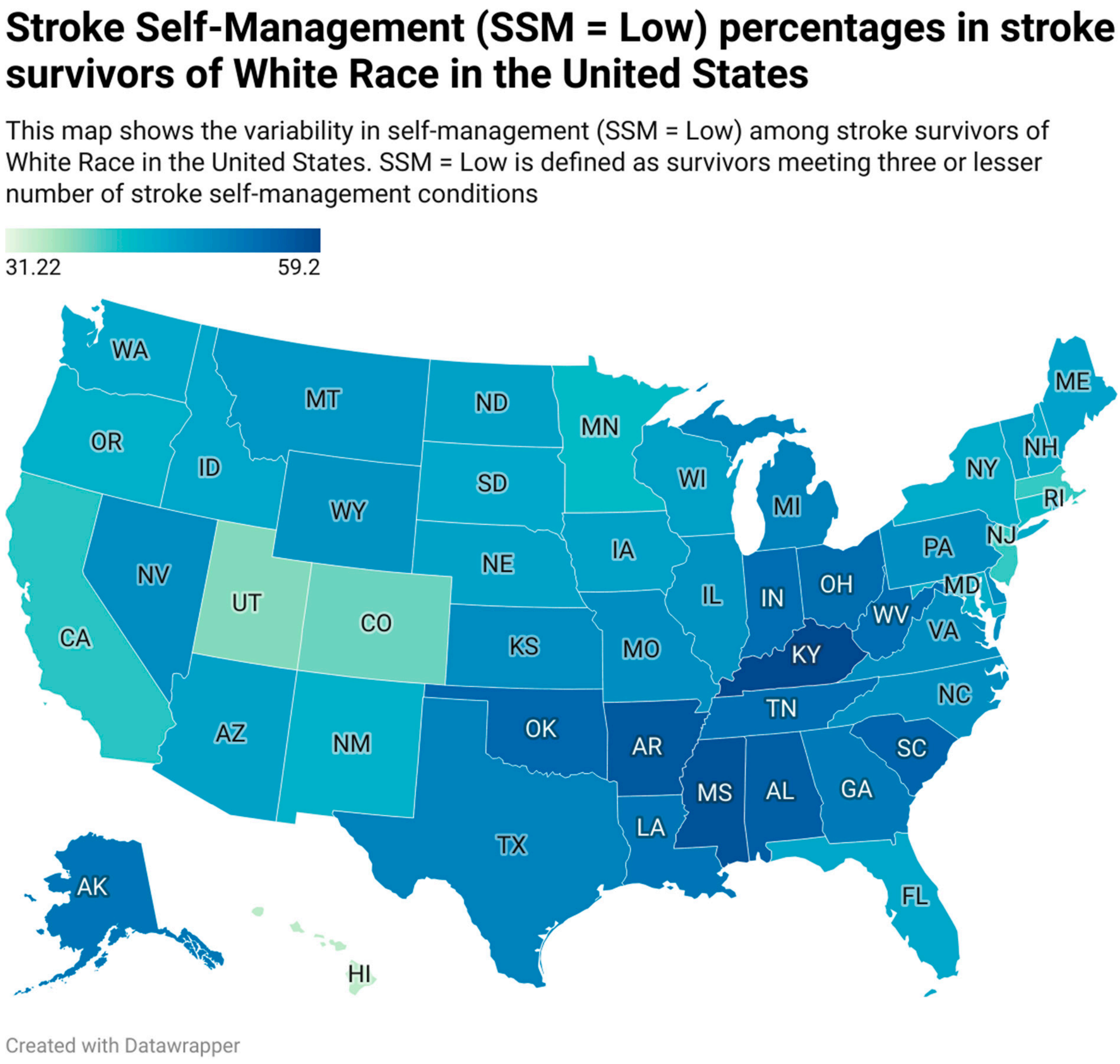

Figure S4: SSM percentages in stroke survivors of Black Race

Stroke Self-Management (SSM = Low) percentages in stroke survivors of Black Race in the United States

This map shows the variability in self-management (SSM = Low) among stroke survivors of Black Race in the United States. SSM = Low is defined as survivors meeting three or lesser number of stroke self-management conditions

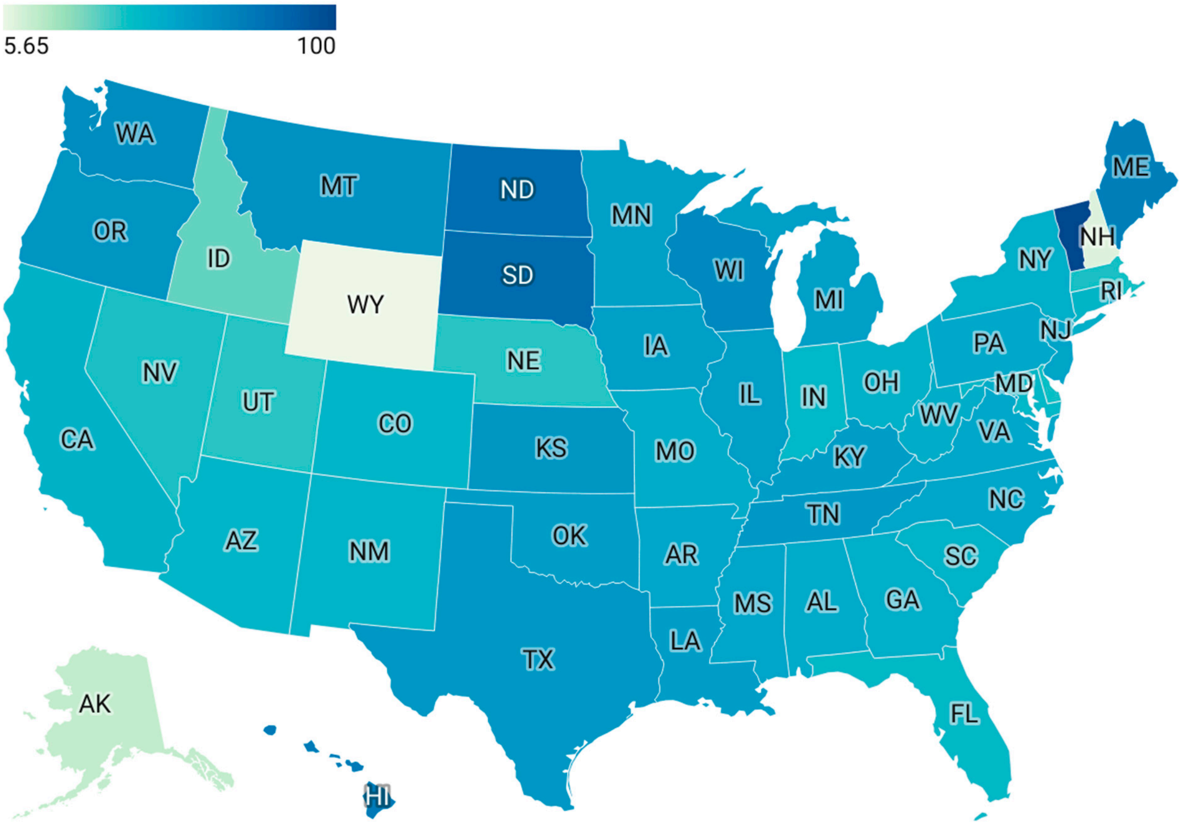

Created with Datawrapper

Figure S5: SSM percentages in stroke survivors of Other Races

Stroke Self-Management (SSM = Low) percentages in stroke survivors of Other Races in the United States

This map shows the variability in self-management (SSM = Low) among stroke survivors of Other Races in the United States. SSM = Low is defined as survivors meeting three or lesser number of stroke self-management conditions

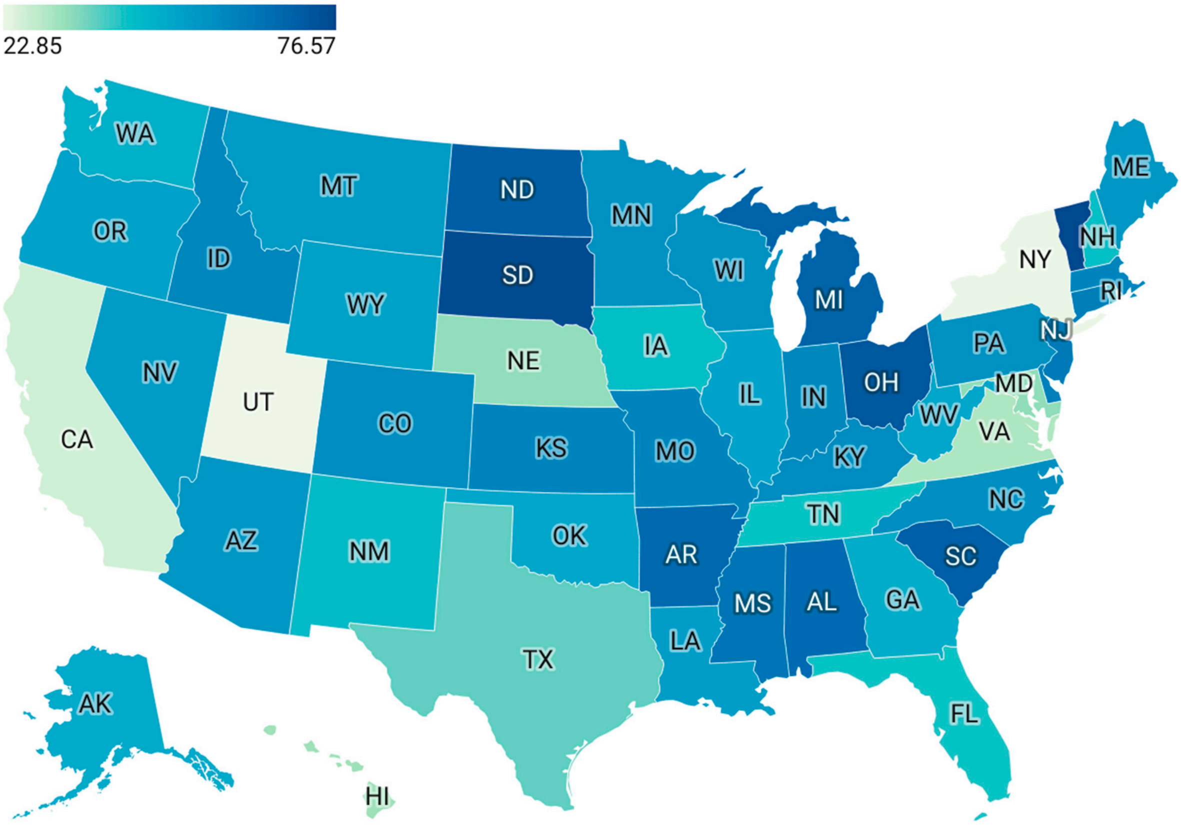

Created with Datawrapper

Figure S6: SSM percentages in stroke survivors of Multi Races

Stroke Self-Management (SSM = Low) percentages in stroke survivors of Multi Race in the United States

This map shows the variability in self-management (SSM = Low) among stroke survivors of Multi Race in the United States. SSM = Low is defined as survivors meeting three or lesser number of stroke self-management conditions

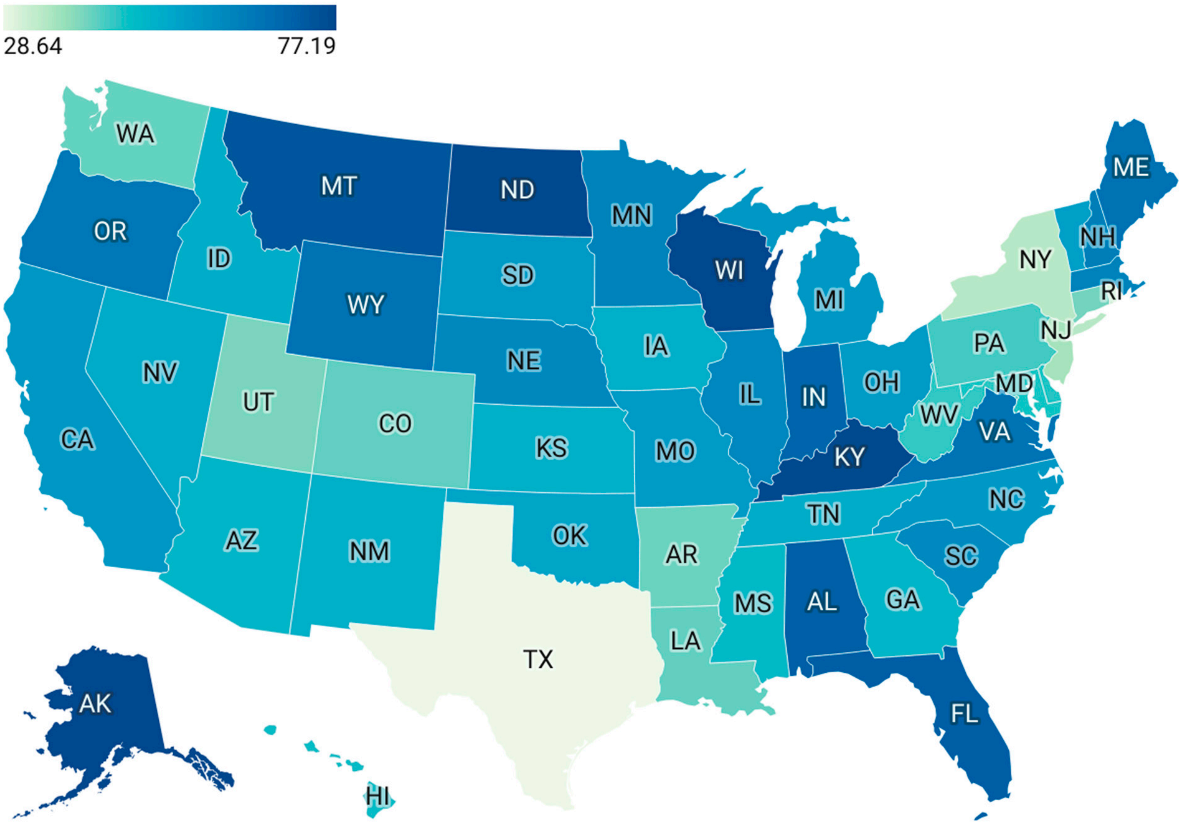

Created with Datawrapper

Figure S7: SSM percentages in stroke survivors of Hispanic Race

Stroke Self-Management (SSM = Low) percentages in stroke survivors of Hispanic race in the United States

This map shows the variability in self-management (SSM = Low) among stroke survivors of Hispanic Race in the United States. SSM = Low is defined as survivors meeting three or lesser number of stroke self-management conditions

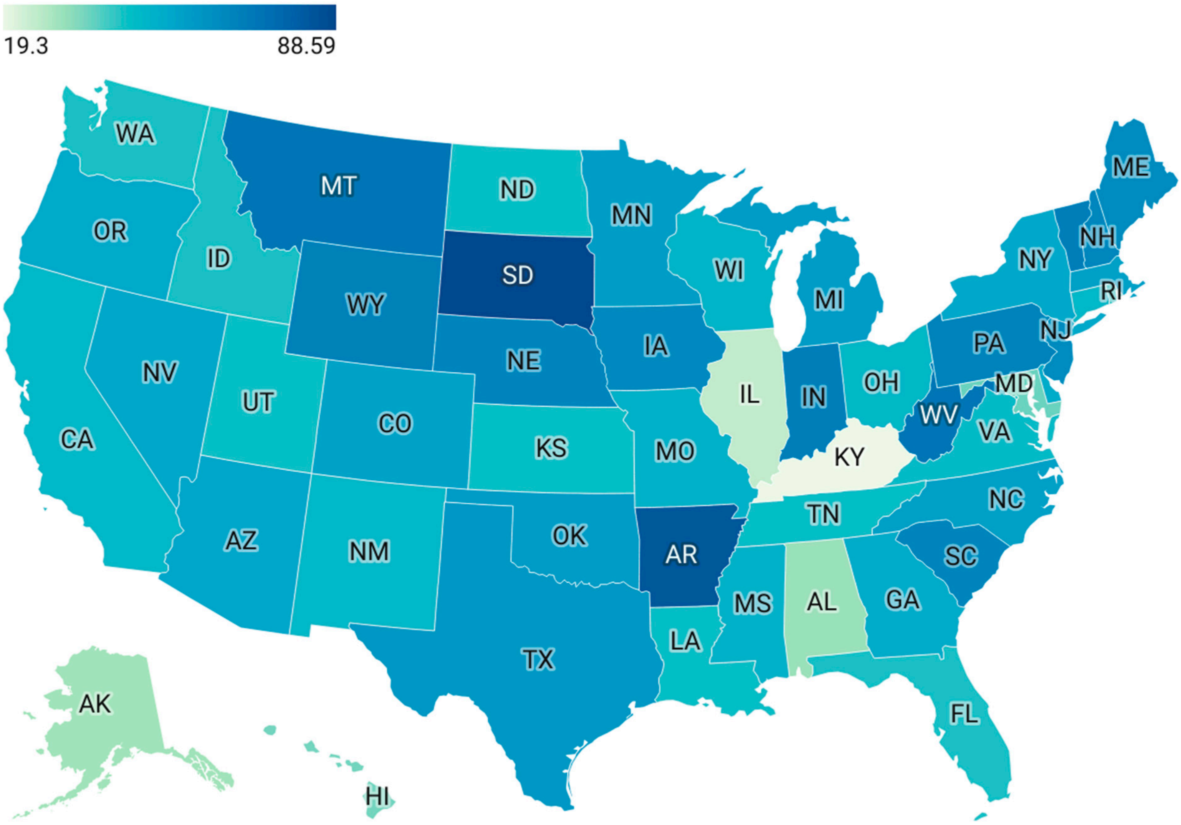

Created with Datawrapper
